# The effect of Baduanjin exercise on the quality of life in patients recovering from COVID-19: A protocol for systematic review and meta-analysis

**DOI:** 10.1097/MD.0000000000022229

**Published:** 2020-09-11

**Authors:** Qian Ma, Zhihua Yang, Feng Zhu, Haojia Chen, Haolin Yang, Shuping Wang

**Affiliations:** a First Teaching Hospital of Tianjin University of Traditional Chinese Medicine; b Graduate School, Tianjin University of Traditional Chinese Medicine, Tuanbo New Town, Tianjin; c Department of Traditional Chinese Medicine, Hebei North University, Zhangjiakou, Hebei Province; d Tianjin University of Traditional Chinese Medicine, Tuanbo New Town; e Characteristic Medical Center of Chinese People's Armed Police Force, Tianjin, P. R. China.

**Keywords:** Baduanjin exercise, COVID-19, systematic review

## Abstract

**Background:**

Since the outbreak of COVID-19, the number of COVID-19 patients has been on the rise. With the improvement of diagnosis and treatment level in various countries, more and more patients have recovered. Baduanjin exercise is a traditional Chinese health care method with a long history, easy-to-learn, and remarkable effect. It is not subject to the constraints of the field and can be practiced at any time. It can be used as an alternative therapy for COVID-19 rehabilitation patients. At present, there are no relevant articles for systematic review.

**Methods:**

We will retrieve a randomized controlled trial of Baduanjin exercise for COVID-19 from the beginning to July 2020. The following databases are areas of concern: Published randomized Cochrane Central Register of Controlled Trials (Central), PubMed, EMBASE, Web of Science, China National Knowledge Infrastructure, Chinese Biomedical Literature Database, and Wan-fang Database-controlled trials in Chinese and English related to Baduanjin exercise and COVID-19 were included. The main result was the effect of Baduanjin exercise on the quality of life in patients recovering from COVID-19. Secondary results to accompany symptoms (such as muscle pain, cough, sputum, runny nose, sore throat, chest tightness, shortness of breath, difficulty breathing, fatigue, headache, nausea, vomiting, anorexia, diarrhea), disappearance rate, 2 consecutive (not on the same day) COVID-19 negative rate of nucleic acid test results, the quality of life improved, improve CT images, the average hospitalization time, severe form of common clinical cure rate and mortality.

**Results:**

The results of this study will provide researchers in the field of COVID-19 with a current synthesis of high-quality evidence.

**Conclusion:**

The conclusion of this study will provide evidence for judging whether Baduanjin exercise is an effective intervention for the quality of life of rehabilitative patients.

**PROSPERO registration number:**

CRD42020199443

## 1 Introduction

Since the middle of December 2019, a new type of coronavirus infection has been prevalent in Wuhan, China, and has rapidly spread to a large area. Coronaviruses (CoVs) belong to the subfamily Orthocoronavirinae in the family Coronaviridae, Order Nidovirales. There are 4 genera within the subfamily Orthocoronavirinae, namely Alphacoronavirus (α-CoV), Betacoronavirus (β-CoV), Gammacoronavirus (γ-CoV), and Deltacoronavirus (δ-CoV).^[1]^ The CoV genome is an enveloped, positive-sense, single-stranded RNA with a size varying between 26 kb and 32 kb, the largest genome of known RNA viruses.^[2]^ Similar to other RNA viruses, this family is characterized by significant genetic variability and high recombination rate that enable them to be easily distributed among humans and animals worldwide.^[3]^ Coronaviruses (CoVs), mainly targeting human respiratory system, are responsible for health-threatening outbreaks including severe acute respiratory syndrome (SARS), Middle East respiratory syndrome (MERS), and lastly coronavirus disease 2019 (COVID-19).^[4]^ The etiological agent of COVID-19 has been confirmed as a novel coronavirus, severe acute respiratory syndrome coronavirus 2 (SARS-CoV-2), which is a highly virulent and pathogenic viral infection, belonging to the coronavirus family with a high mutation rate. COVID-19 generally has an incubation period of 2 to 14 days and is transmitted by respiratory infection or contact with infected droplets. The initial host may have been bats, as genome analysis has shown that SARSCOV-2 is systemically genetically related to SARS-like bat viruses.^[2]^ People are generally susceptible to infection. The most common symptoms at onset of COVID-19 illness are fever, cough, and fatigue, while other symptoms include sputum production, headache, hemoptysis, diarrhoea, dyspnoea, and lymphopenia.^[5–8]^ As of August 8, 2020, more than 19 million people have been infected worldwide. This is a terrible figure that poses a huge challenge to global health and brings huge losses to the global economy.^[9]^ Currently, the sequelae of COVID-19 patients are rarely reported, but the impact on people's quality of life is inevitable, especially in people with COVID-19 sequelae. COVID-19, as a sudden outbreak, pandemic infectious disease, causes an emotional response of extreme fear and uncertainty in the public, which often leads to negative social behavior and may involve public mental health issues such as anxiety, depression, insomnia, aggression, depression, and hysteria.^[10]^ The COVID-19 pandemic is having serious consequences for the mental health of the population, and the consequences are considered very bad.^[11]^ Given the huge physical and psychological damage COVID-19 has caused to human beings and the lack of good therapeutic interventions, a simple, reliable, and feasible treatment is urgently needed to improve the physical and mental health of the public. Baduanjin exercise is a traditional Chinese movement method with a long history. It has the function of keeping in good health. It is a means of prevention and recovery therapy.^[12]^ As an adjuvant therapy and rehabilitative method, Baduanjin exercise is effective, safe, and noninvasive. It is beneficial to the quality of life, sleep quality, balance, grip strength, trunk flexibility, systolic and diastolic blood pressure, and resting heart rate.^[1]^ It also plays a role in reducing depression and anxiety symptoms in patients with psychosomatic illnesses.^[13]^ It can improve cognitive function in different age groups and different clinical groups, as well as psychological and physiological parameters.^[14]^ It has the characteristics of good analgesic effect, no side effects, convenient operation, low economic burden, and more conducive to promoting the rehabilitation of patients. At present, there is still a lack of evidence-based medicine in the treatment of COVID-19 patients in rehabilitation, and it is very necessary to improve anxiety, tension, and other negative emotions of patients in rehabilitation and improve their quality of life. Therefore, it is necessary to review it and provide evidence for clinicians.

## 2 Methods

### 2.1 Study registration

The systematic review protocol has been registered in PROSPERO. The registration number: CRD42020199443, the consent of this protocol report is based on the Preferred Reporting Items for Systematic Reviews and Meta-Analyses Protocols (PRISMAP) statement guidelines.^[15]^

### 2.2 Inclusion criteria for study selection

#### 2.2.1 Type of study

We will include articles related to Baduanjin exercise therapy of patients recovering from COVID-19. Due to language restrictions, we will search for articles in English and Chinese in order to get a more objective and true evaluation, all articles included are randomized controlled trial (RCT) type articles.

#### 2.2.2 Type of participant

All COVID-19 survivors regardless of age, sex, weight, race, education, or economic status. Pregnant women, postoperative infections, psychiatric patients, patients with severe cardiovascular, and hepatorenal diseases were not included.

#### 2.2.3 Type of intervention

Baduanjin exercise is a traditional Chinese exercise method, while other traditional Chinese medicine treatments such as acupuncture, massage, cupping, and traditional Chinese medicine will be excluded. We will compare the following interventions: treatment other than the Baduanjin exercise (e.g., routine or standard care, placebo, wait-list controls).

#### 2.2.4 Type of outcome measure

This paper mainly studied the effect of Baduanjin exercise on the quality of life of convalescent patients. The main result was the effect of Baduanjin exercise on the quality of life of convalescent patients. Secondary results to accompany symptoms (such as muscle pain, cough, sputum, runny nose, sore throat, chest tightness, shortness of breath, difficulty breathing, fatigue, headache, nausea, vomiting, anorexia, diarrhea), disappearance rate, 2 consecutive (not on the same day) COVID - 19 negative rate of nucleic acid test results, the quality of life improved, improve CT images, the average hospitalization time, severe form of common clinical cure rate, and mortality.

### 2.3 Data sources

From the beginning to July 2020, we will retrieve the following electronic databases: Cochrane Central Register of Controlled Trials (Central), PubMed, EMBASE, Web of Science, China National Knowledge Infrastructure, Chinese Biomedical Literature Database, And Wan Fang - Database. For other sources, we also plan to manually search for unpublished conference articles and bibliographies of established publications.

### 2.4 Search strategy

The search keywords on PubMed are as follows: Baduanjin exercise (such as “Baduanjin Qigong,” “Qigong,” “health preservation,” and “rehabilitation”); COVID-19 (such as “Corona Virus Disease 2019” or “Corona Virus”); convalescence (e.g., “rehabilitation” or “convalescent period” or “decubation”); randomized controlled trials ((e.g., “randomized” or“ randomly” or “clinical trial”). Combinations of Medical Subject Headings (MeSH) and text words will be used. The same search term is used in electronic databases in China. These search terms are shown in Table 1.

**Table 1:**
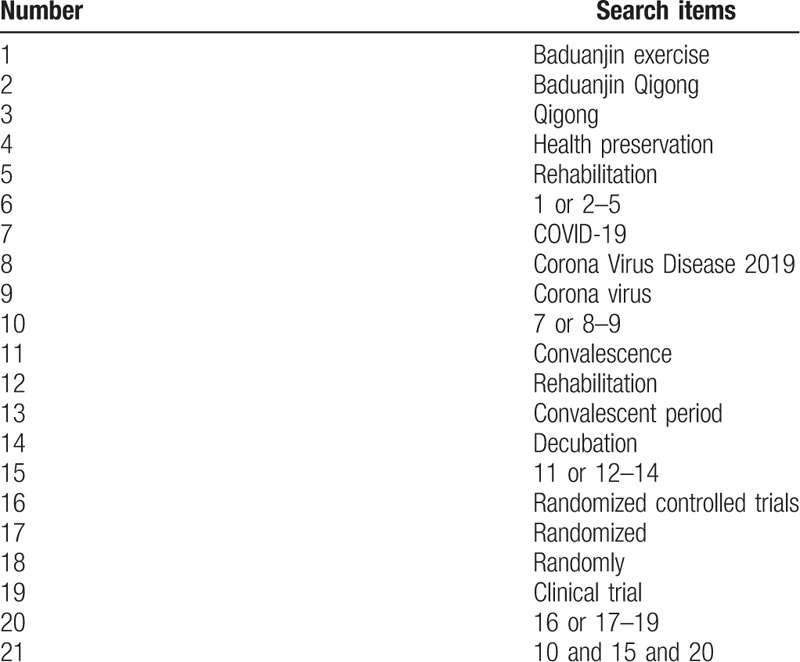
Search strategy for the PubMed database.

### 2.5 Data collection and analysis

#### 2.5.1 Selection of studies

**Figure 1:**
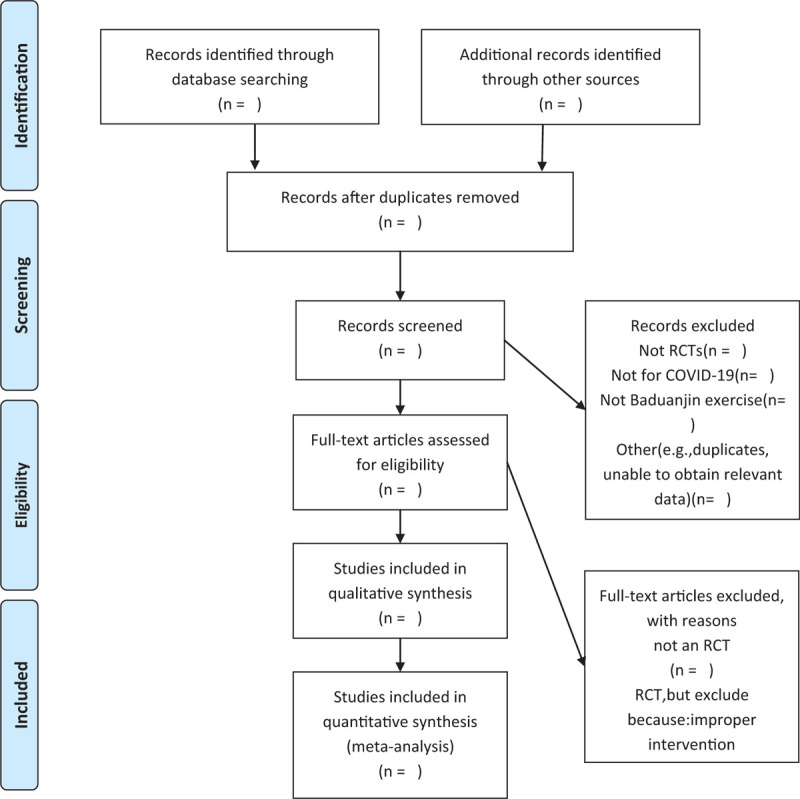
Flow chart of the study.

We chose the PRISMA flow chart to show the process of selecting literature for the entire study (Fig. 1). Before searching the literature, all reviewers will discuss and determine the screening criteria. After the screening requirements are clearly defined, the 2 reviewers (QM and ZY) will independently review and screen the literature. They screened the titles and abstracts of the literature, in order to get qualified studies, and then excluded some duplicate studies or studies with incomplete information. We will also try to obtain the full text, and the obtained literature will be managed by using EndNote software, V.X8 (United States). In case of disagreement between the 2 reviewers, discussions will be held with the third author (FZ) for arbitration.

#### 2.5.2 Data extraction and management

The authors will strictly follow the inclusion criteria and select RCT articles related to the topic. Through the analysis of the article, we know participants’ characteristics (gender, age, height, weight, BMI), interventions, outcomes, the study characteristics (press, nationality, journals, research design), adverse reactions, etc. If there is any disagreement between the 2 authors in the literature data extraction, a third article participant (FZ) will discuss the decision together. If there is a lack of data in the literature, we will contact the author or publisher as much as possible.

#### 2.5.3 Assessment of risk of bias in included studies

We will use the Cochrane collaborative tool to independently assess the risk of bias in the included studies. We will evaluate the following aspects of the article: sequence generation, assignment sequence hiding, blindness of participants and staff, outcome evaluators, incomplete result data, selective result reporting, and other sources of bias. The risk of bias is evaluated at 3 levels, namely, low risk, high risk, and ambiguity. If the information is vague, we will try to contact the author of the article.

#### 2.5.4 Measures of treatment effect

In this protocol, we will use 95% confidence interval (CI) risk ratio (RR) to rigorously analyze the dichotomous data. And for the continuous data, mean difference (MD) or standard MD is used to measure the efficacy of 95% CI.

#### 2.5.5 Unit of analysis issues

We will include data from parallel group design studies for meta-analysis. In these trials, we will collect and analyze individual measurements of each outcome for each participant.

#### 2.5.6 Management of missing data

We will try our best to ensure the integrity of the data. If the included RCT data is not complete, we will try every means to contact the corresponding author of the article, including sending emails or making a phone call. If the corresponding author cannot be contacted, we will remove the experiment with incomplete data. After data integrity is assured, intention analysis therapy and sensitivity analysis will be performed.

#### 2.5.7 Assessment of heterogeneity

For the detection of heterogeneity, the I^2^ test will be used to detect the heterogeneity among trials. When the I^2^ test value is <50% and *P* value > 1, we think there is no heterogeneity between these trials, and when the I^2^ test value is >50% and the *P* value is <1, there is significant heterogeneity between these included trials. If significant differences are detected, we will analyze the possible causes of heterogeneity, and then we can use the random effects model.

#### 2.5.8 Assessment of reporting biases

In this analysis, once >10 trials are included, funnel plots could be used to test for reporting bias.

#### 2.5.9 Data synthesis

We will use Review Manager Software (RevMan) V.5.3 (Copenhagen, Denmark) for data analysis and quantitative data synthesis. If there is no finding of statistical heterogeneity, the fixed-effect model is used for data synthesis. If there is significant statistical heterogeneity, we will use the random effect model, and all participants will explore the possible causes from a clinical and methodological perspective and provide a descriptive or subgroup analysis.

#### 2.5.10 Subgroup analysis

Subgroup analysis will be performed to explain heterogeneity if possible. Factors such as different types of control interventions and different outcomes will be considered.

#### 2.5.11 Sensitivity analysis

Based on sample size, study design, heterogeneous quality, methodological quality, and statistical model, sensitivity analysis will be performed to exclude trials with quality defects and ensure the stability of the analysis results.

#### 2.5.12 Grading the quality of evidence

This paper will use the evidence quality rating method to evaluate the results obtained from this analysis. GRADE is generally applied to a large amount of evidence. It has 4 evaluation levels, namely, high, medium, low, and very low. GRADE was used to evaluate the bias, inconsistencies, discontinuities, and inaccuracies of test results. In the context of the system review, quality reflects our confidence in the effectiveness of assessment.^[16]^

#### 2.5.13 Ethical review and informed consent of patients

Ethics and dissemination: The content of this article does not involve moral approval or ethical review and will be presented in print or at relevant conferences.

## 3 Discussion

As the number of people cured of COVID-19 increases, a large number of people will enter the recovery phase. People at this stage are often accompanied by a series of symptoms such as anxiety and insomnia. Evidence supports the efficacy and safety of Baduanjin exercise for anxiety, insomnia, and pain relief.

This review is divided into 4 parts: identification, literature inclusion, data extraction, and data synthesis. Systematic review of RCT literature; this paper will evaluate the efficacy of Baduanjin exercise in the treatment of COVID-19 convalescent patients. Our study also has limitations. The language bias here is that we only search Chinese and English literature. This study can provide a basis for clinicians to choose alternative therapy for further research.

## Acknowledgments

This study was supported by Danshenyin prevents and treats hyperlipidemia model mice; research on related mechanisms (JYT2019010).

## Author contributions

**Conceptualization:** Qian Ma.

**Data curation:** Zhihua Yang, Haojia Chen, Haolin Yang, Feng Zhu.

**Formal analysis:** Haolin Yang, Feng Zhu.

**Resources:** Shuping Wang, Feng Zhu.

**Software:** Zhihua Yang.

**Writing – original draft:** Qian Ma.

**Writing – review & editing:** Zhihua Yang, Haojia Chen, Shuping Wang.
